# Placement of Plastic Stents after Direct Endoscopic Necrosectomy through a Novel Lumen-Apposing Metal Stent for Effective Treatment of Laterally Extended Walled-Off Necrosis: A Pilot Study

**DOI:** 10.3390/jcm12031125

**Published:** 2023-01-31

**Authors:** Kyong Joo Lee, Se Woo Park, Da Hae Park, Jung Hee Kim, Jang Han Jung, Dong Hee Koh, Jin Lee, Mi Gang Kim

**Affiliations:** 1 Division of Gastroenterology, Department of Internal Medicine, Hallym University Dongtan Sacred Heart Hospital, Hallym University College of Medicine, Hwaseong 18450, Republic of Korea; 2 Health Care Center, Hallym University Dongtan Sacred Heart Hospital, Hallym University College of Medicine, Hwaseong 18450, Republic of Korea

**Keywords:** laterally extended, walled-off necrosis, endoscopic necrosectomy, plastic stent, lumen-apposing metal stent

## Abstract

Direct endoscopic necrosectomy (DEN) using a lumen-apposing metal stent (LAMS) is a standard therapy for the management of symptomatic walled-off necrosis (WON). Here, we demonstrated the efficacy of the routine placement of long plastic stents after a DEN session to treat laterally extended WON. Patients (n = 6) with symptomatic laterally extended WON who underwent DEN after long plastic stent placement were included. The primary endpoint was clinical efficacy of the procedure. The technical and clinical success rates were 100% without major adverse events. The WON extended to the pelvic cavity or pericolic area, and the WON size was between 18.6 and 35.8 cm in length. The median number of DEN sessions was 10 (range 6–16), and two or three long plastic stents were placed after every DEN session. Only one patient suffered from pneumoperitoneum during DEN, which spontaneously resolved within 20 min. Placement of a long plastic stent after DEN using LAMS is a minimally invasive and effective treatment for symptomatic laterally extended WON. Further studies are needed to define the indications and most suitable patients.

## 1. Introduction

Endoscopic ultrasonography (EUS)-guided transmural drainage is the standard treatment modality for the management of symptomatic pancreatic fluid collection (PFC) [1]. Walled-off necrosis (WON) mainly contains solid components such as pancreatic necrosis within a mature encapsulated wall; therefore, transmural drainage alone may not be sufficient, and additional direct endoscopic necrosectomy (DEN) may be required [2,3,4]. Generally, DEN through the stent includes the drainage of liquid materials and endoscopic debridement of solid debris from the WON through the gastric or duodenal lumen. Recent studies [5,6] reported that the endoscopic approach is minimally invasive and provides superior outcomes with fewer adverse events and lower medical costs than surgical necrosectomy for symptomatic WON. However, EUS-guided PFC drainage with a DEN through the stent has limitations in laterally placed WON that are far from the stomach or duodenum, and has traditionally been treated by a surgical approach [7]. A recent study demonstrated that sinus tract endoscopy (STE) is an effective alternative method for treating laterally placed WON, which is not feasible for DEN. However, the technique of STE, which includes percutaneous drainage and retroperitoneal endoscopic necrosectomy, leads to inconvenience, painful discomfort, and a potential risk of pancreaticocutaneous fistula formation. Lavage of the WON via a nasocystic catheter is an alternative method to continuously wash out the debris and maintain stent patency [8]. As another tool for laterally extended WON, video-assisted retroperitoneal debridement (VARD) has been considered a suitable surgical approach for patients with a central distribution of necrosis that extends down into the right or left paracolic gutter [1]. Consequently, a recent consensus based on expert opinions recommended that aggressive percutaneous drainage, with or without surgical approaches, should be applied to WON with large paracolic gutter extension.

Nevertheless, we hypothesized that an endoscopic approach including EUS-PFC drainage with a lumen-apposing metal stent (LAMS) following multiple sessions of DEN may effectively be applied to laterally extended WON. Furthermore, placing multiple plastic stents after each session of DEN to the deep portion of the WON can prevent tract narrowing and secondary infection. However, no study has yet reported the routine placement of long plastic stents after DEN sessions for the effective endoscopic treatment of laterally extended WON; therefore, we aimed to evaluate its efficacy.

## 2. Methods

### 2.1. Patients

The endoscopy databases at our hospital were retrospectively queried for all patients who underwent EUS-PFC drainage of a WON between February 2014 and July 2022. We included patients who had WON confirmed by computed tomography (CT) with the following conditions: (1) refractory abdominal pain, (2) gastric outlet or duodenal obstruction, (3) biliary obstruction, (4) rapidly growing size, and/or (5) infection symptoms or signs. According to the Revised Atlanta Classification [3], a WON is defined as a mature collection of peripancreatic necrosis encapsulated by a well-defined inflammatory wall. Among them, patients who had laterally extended WON, which extended to the paracolic gutter or pelvic cavity from the stomach or duodenum, were finally included. All patients underwent DEN several times after EUS-PFC drainage with a LAMS, which enabled free passage of the gastroscope. We reviewed the data on patient demographics, endoscopic findings, clinical outcomes, and procedure-related adverse events.

### 2.2. Endoscopic Procedures

**Figure 1:**
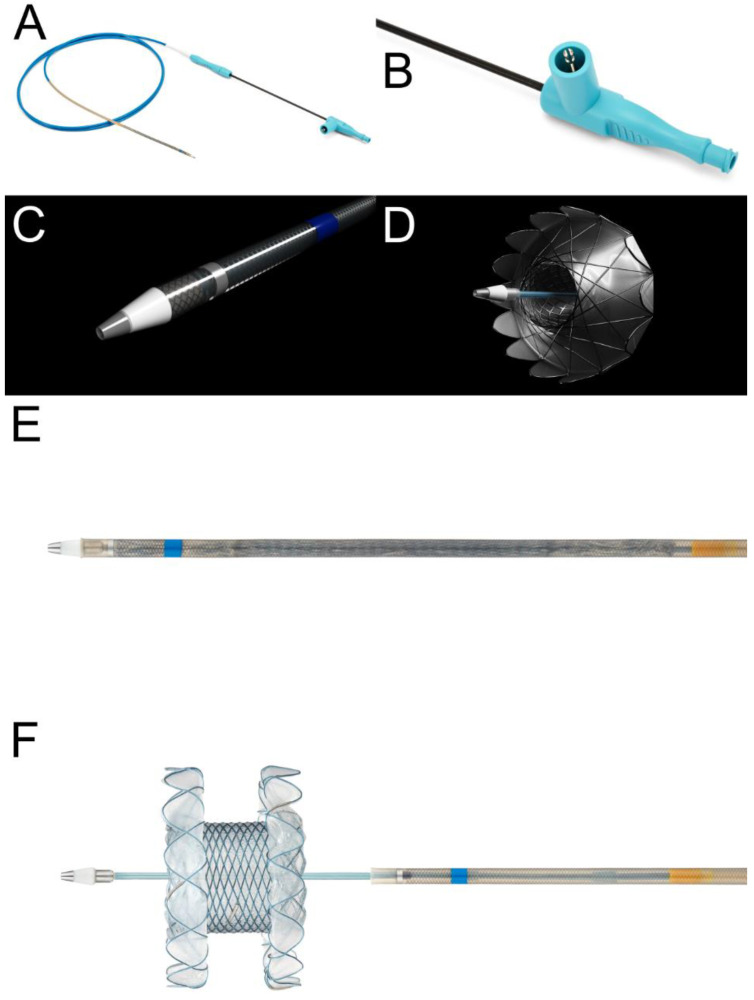
Real photos and illustrations of the novel lumen-apposing metal stent (LAMS) (Niti-S HOT SPAXUS^TM^, Taewoong Medical, Gyeonggi-do, Republic of Korea). (**A**) This stent is a fully covered self-expandable metal stent preloaded with the Hot SPAXUS™ Delivery System. (**B**) The delivery system allows endoscopic control and employs a locked two-step release system to prevent unintended deployment of the proximal flange. (**C**) There was a blue marker on the outer sheath to confirm complete deployment of the distal flange. (**D**) After half-deployment, inner anchoring flanges can be folded back to hold the inner luminal interfaces for apposition. (**E**) It has a through-the-scope electrocautery-enhanced delivery system compatible with therapeutic echoendoscopes, having a working channel of 3.7 mm diameter or larger. (**F**) The stent has bilateral anchor flanges to provide lumen-to-lumen anchoring. These features reduce the risk of stent migration and leakage alongside the stent, as well as prevent tissue in-growth and enable easy removal.

**Figure 2:**
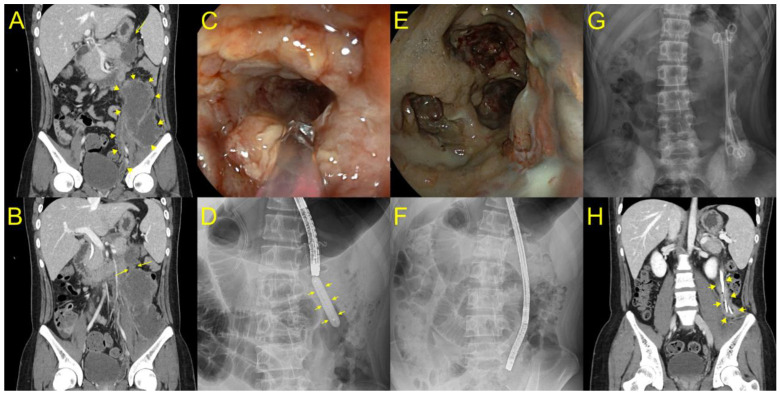
Placement of long plastic stents to the deep portion of walled off necrosis (WON) after direct endoscopic necrosectomy (DEN) for endoscopic treatment of laterally extended WON (yellow arrow) (**A**). (**B**) Computed tomography showing a huge WON extended to the left pelvic cavity communicating to a small portion of the necrotic cavity concatenated with the stomach through the narrow neck (yellow arrow) portion. (**C**) A gastroscope was inserted into the proximal portion of WON through a lumen-apposing metal stent (LAMS), and we found a luminal stricture of the neck portion of WON. (**D**) Balloon dilatation (yellow arrow) was performed for deep insertion of an endoscope to the laterally extended WON. (**E**,**F**) The cavity was lavaged with normal saline, and necrotic debris was progressively removed with snare and grasping forceps. (**G**) After DEN, three plastic stents with double pigtail configuration were fixed in place through the LAMS to the deep portion of WON until the next session of DEN. (**H**) After 16 DEN sessions, where the long plastic stents remained in the deep portion of WON, the patient recovered with complete WON resolution (yellow arrow).

All procedures were performed using a linear array echoendoscope (EG-530 UT2, Fujifilm Co., Tokyo, Japan) by an experienced echoendoscopist (S.W.P) with a current volume of ≥100 EUS-guided interventions per year using a well-established technique [9]. After positioning the lesion in the natural path of the expected needle track, the operators confirmed the absence of intervening vasculature using a color doppler. A 19-gauge standard aspiration needle (EZ Shot 3, Olympus Medical, Japan) was inserted into the WON. After the aspiration of adequate amounts of fluid, the same dose of contrast material was injected into the WON. A 0.035-inch guidewire (OptimosTM Guidewire, Taewoong Medical, Goyang, Republic of Korea) was then advanced into the collection and coiled under fluoroscopic guidance. Subsequently, the needle was removed. The electrocautery-enhanced tip of the LAMS was then placed over a guidewire and advanced into the WON with current using an electrosurgical unit (Auto Cut mode, 80–120 Watts, 400–500 Vp). A novel LAMS (Niti-S Hot SPAXUS Stent™, Taewoong Medical, Goyang, Republic of Korea), which is a fully covered self-expanding stent with bilateral anchor flanges to provide lumen-to-lumen anchoring, was used in all patients. These features reduce the risk of stent migration and leakage alongside the stent, prevent tissue growth, and enable easy removal (Figure 1). In this study, a 16 mm diameter was preferred because a larger diameter would allow access to the cavity for better clearance of necrotic debris and future DEN. Thereafter, patients who had persistent symptoms without clinical improvement underwent DEN according to the step-up approach [10]. For DEN, a gastroscope or colonoscope was transmurally entered into the necrotic cavity for debridement using snares or forceps at regular intervals until complete resolution of the WON was confirmed endoscopically or radiologically. After each DEN session, two or more 7-Fr double-pigtail plastic stents (Zimmon^®^ Biliary Stent, Cook Medical, Bloomington, IN, USA) were placed into the deep cavity at the paracolic gutter or pelvic cavity of the WON for effective drainage and to maintain a track for endoscope passage (Figure 2).

### 2.3. Outcome Measurements and Definitions

The primary endpoint was to assess the clinical efficacy of DEN following plastic stent placement through the LAMS for the treatment of laterally extended WON. Clinical improvement was defined as complete clinical and radiological resolution of WON without requiring further percutaneous or surgical treatment [11]. The secondary endpoints were technical success rate, removability of the LAMS, and immediate or delayed adverse events.

### 2.4. Ethics Approval

The study was conducted in accordance with the protocol of the Declaration of Helsinki and was approved by the Institutional Review Board of the Ethics Committee of Hallym University Dongtan Sacred Heart Hospital (IRB file no: 2022-01-013). As this was a retrospective analysis, the need for informed consent was waived.

## 3. Results

### 3.1. Patients’ Baseline Characteristics

The patient characteristics are summarized in Table 1. During the study period, 112 patients who underwent EUS-PFC for pseudocysts or WON were eligible for this study. Among them, 106 were excluded for the following reasons: patients with pseudocysts (n = 81), patients with isolated WON located near the stomach or duodenum (n = 21), and patients in whom endoscopic treatment alone was not possible for the complete resolution of WON; therefore, required additional modalities such as surgical approaches (e.g., VARD) or percutaneous drainage (n = 4). Finally, six patients (two men; median age, 49 years; range, 16–76 years) were included in the analyses, and their clinical outcomes were evaluated. The etiologies of acute pancreatitis were gallstones (two patients), hypertriglyceridemia (two patients), and post-endoscopic retrograde cholangiopancreatography (two patients). The WON was located in addition to the pancreatic body/tail in three cases, diffusely from head to tail in one case, and in the pancreatic head in one case. According to the location, EUS-PFC drainage was performed through the stomach (five cases) and the duodenum (one case). Three WON cases extended to the pelvic cavity, one to the left paracolic gutter, one to the right paracolic gutter, and one to both paracolic gutters. The largest WON size was 35.8 cm, while the smallest size was 18.6 cm by longitudinal length. Regarding their symptoms, all were suffering from abdominal pain and fever.

### 3.2. Procedure Description

EUS-PFC drainage was achieved using the novel LAMS (NITI-S HOT SPAXUS Stent™, 16 mm in diameter and 20 mm in length) in all six cases. Despite EUS-PFC drainage using LAMS, all patients had persistent signs of infection, such as abdominal pain and fever, as the WON mainly had solid necrosis. Therefore, all the patients received antibiotic therapy with carbapenems. Considering the persistent symptoms, DEN was performed through LAMS using a gastroscope or colonoscope under CO_2_ insufflation. DEN was repeated every two days depending on the endoscopic presentation of necrosis, and until most of the non-adherent necrotic material was pushed out, clinical improvement was achieved. These necrosectomy sessions were performed using standard 15-mm snares, grasping forceps, or suction with normal saline flushing using a water-jet system. The DEN duration was limited to 30 min for every session. After each DEN session, two or three long plastic stents (15 cm) with a double pigtail configuration remained in place through the LAMS to the deep portion of the WON until the next DEN session. An exemplary case of the entire procedure is shown in Video S1. After endoscopic and clinical improvement, the plastic stents and LAMS were removed endoscopically without difficulty in all cases.

### 3.3. Outcomes

The technical success rate was 100%, and clinical success was achieved in all patients (6/6), with complete removal of non-adherent necrotic debris and clinical improvement. The median number of DEN sessions was 10 (range 6–16). In one case, a large pneumoperitoneum occurred during DEN. However, the free air in the peritoneum was completely absorbed within 20 min. No other major adverse events, such as significant bleeding or overt perforation, were observed. In all cases, no further percutaneous or surgical treatment was required. All patients who underwent DEN following plastic stent placement were symptom-free for at least three months.

## 4. Discussion

**Figure 3:**
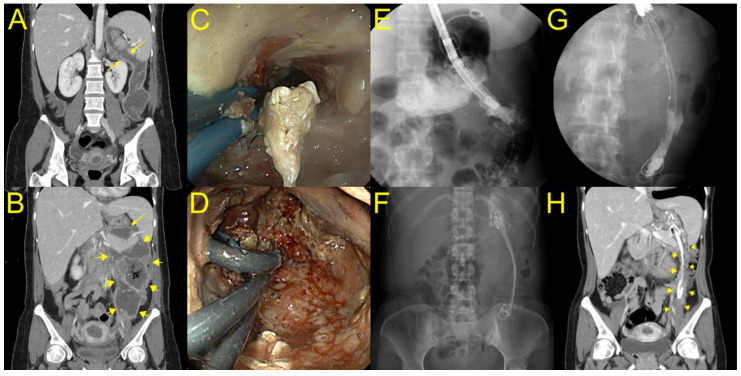
A case of elongated WON extended to the left paracolic gutter. (**A**,**B**) Computed tomography showing that an elongated WON (yellow arrow) extended to the left paracolic gutter was concatenated with the stomach. (**C**–**E**) A gastroscope was inserted into the proximal portion of the cavity through a lumen-apposing metal stent (LAMS), and we found a long narrowing WON endoscopically and radiologically. (**F**) After DEN, three plastic stents with double pigtail configuration remained in place through LAMS to the deep portion of WON until the next session of DEN. (**G**) After nine DEN procedures, long plastic stents remained in the deep portion of WON with partial improvement radiologically. (**H**) After 11 DEN sessions, where the long plastic stents remained in the deep portion of WON, the patient recovered with complete WON resolution (yellow arrow).

This is the first case series to demonstrate successful endoscopic treatment of laterally extended WON with DEN, followed by the placement of multiple plastic stents. The first report of endoscopic transmural therapy for pancreatic necrosis was described by Baron et al., who used a plastic stent with nasocystic irrigation to treat WON [12]. However, to date, there is no study reporting on the use of long plastic stents after EUS-PFC drainage with LAMS following multiple DEN sessions for the treatment of laterally extended WON. In the current study, we demonstrated that additional placement of two or three long plastic stents with a double pigtail shape through the LAMS up to the deep portion of the WON can modulate persistent drainage, even in severe cases of laterally extended WON, and minimize the risk of migration. Although a major limitation of plastic stents is the frequent occlusions due to their small-caliber diameter, long plastic stents can prevent further narrowing of the track according to the healing process with regenerative tissue hyperplasia in cases with an elongated cavity (Figure 3). Consequently, plastic stents allow for the endoscopic track to be maintained in the WON and facilitate the subsequent DEN. In one patient with an elongated WON cavity and a narrow neck portion, pneumoperitoneum occurred during DEN using grasping forceps, and the patient recovered without any further interventions. In this case, only a small cavity volume concatenated with the stomach, connected with a large volume of the majority WON, extended to the pelvic cavity through the narrow neck portion. Therefore, balloon dilatation was performed for easy endoscopic passage at every DEN session in the laterally extended WON (Figure 2).

Based on our experience, DEN for a laterally extended WON can be effective if the major axis of the WON is parallel to the axis of the LAMS and straightens the alignment of the endoscope, which can produce maximal pushing and withdrawal forces. However, if the major axis of the WON and endoscope, as well as the long axis of the LAMS, are not in a straight line, DEN can be performed under sharp angulation of the endoscope. Eventually, endoscopic loop formation can lead to injury to the WON wall and perforation. Additionally, the endoscope cannot reach the target lesion within the WON owing to paradoxical phenomena related to the cane or J-shaped loop formation of the endoscope. In this situation, placing long plastic stents into the deep WON, which the endoscope cannot reach easily, can help to maintain the track, and drain constantly. Therefore, a long plastic stent placement has a synergistic effect on DEN treatment and minimizes invasive interventions.

We strongly support this idea and believe that our results demonstrate effective treatment of laterally extended WON using long plastic stent placement following DEN. Laterally extended WON is a novel indication for the placement of a long plastic stent following DEN sessions. As a first attempt at an endoscopic treatment of laterally extended WON, it has the potential to prevent the morbidity and mortality associated with surgical debridement of patients with multiple organ dysfunctions or failure to thrive. Additionally, novel developments of LAMS in terms of the window for DEN will increase the potential and feasibility of this promising technique.

There are several limitations in this study. First, we did not perform continuous lavage through a nasocystic catheter even though it has been associated with better clinical outcomes resulting from the natural drainage of necrotic debris. However, in our study, the main purpose of placement of additional long plastic stents within the LAMS up to the deep portion of the WON was to prevent further narrowing of the track according to the healing process, as well as persistent internal drainage through and along the plastic stents. In addition, a nasocystic catheter would decrease patients’ quality of life and make them more uncomfortable. Second, this study was a retrospective study and included a small number of patients. A large prospective randomized study is needed to confirm the efficacy of long plastic stent placement following DEN. To that effect, exit strategies such as percutaneous or surgical debridement should be considered in difficult cases where the WON is far from and with no contact to the stomach or duodenum.

## 5. Conclusions

Long plastic stent placement with a combination of DEN through a novel LAMS is minimally invasive, feasible, safe, and has the potential to modulate good clinical outcomes in infected, laterally extended WON. Further studies are needed to define clear indications and determine whether our technique best suits patients with a central distribution of necrosis that extends down into the paracolic gutter or even the pelvis.

## Figures and Tables

**Table 1:** Baseline characteristics of the included patients.

Number of Patients	1	2	3	4	5	6
Patient sex and age, years	Female, 76	Female, 59	Male, 66	Female, 45	Female, 27	Male, 16
Pancreatitis etiology	Post-ERCP pancreatitis	Post-ERCP pancreatitis	Gallstone	Hypertriglyceridemia	Hypertriglyceridemia	Gallstone
Site of WON	Body to tail	Head	Body to tail	Head to tail	Body to tail	Body to tail
Extent of WON	to pelvic cavity	to right pericolic area beside the appendix	to left pericolic area	to both pericolic area	to pelvic cavity	to pelvic cavity
Site of cysto-enterostomy	Stomach	Duodenal 2nd portion	Stomach	Stomach	Stomach	Stomach
Size of WON (cm)	35.8 × 14.9	21.3 × 12.4	18.6 × 13.4	32.1 × 23.7	27.3 × 20.9	32.4 × 10.2
Technical success	Yes	Yes	Yes	Yes	Yes	Yes
Used LAMS (diameter × length)	HOT SPAXUS (16 mm × 20 mm)	HOT SPAXUS (16 mm × 20 mm)	HOT SPAXUS (16 mm × 20 mm)	HOT SPAXUS (16 mm × 20 mm)	HOT SPAXUS (16 mm × 20 mm)	HOT SPAXUS (16 mm × 20 mm)
Total number of DEN sessions after stent placement	6	8	9	14	11	16
Balloon dilatation for narrow track inside WON	No	Yes	No	Yes	No	Yes
Number of plastic stents	2	2	2	3	3	3
Length of plastic stent (cm)	15	12	12	15	15	15
Clinical success	Yes	Yes	Yes	Yes	Yes	Yes
Removability of LAMS	Yes	Yes	Yes	Yes	Yes	Yes
Procedure-related adverse events	No	Incomplete expansion of inner flanges of the LAMS	No	No	No	Pneumoperitoneum during DEN

WON, walled off necrosis; LAMS, lumen-apposing metal stent; DEN, direct endoscopic necrosectomy; ERCP, endoscopic retrograde cholangiopancreatograph.

## Data Availability

The datasets used and analysed during the current study are available from the corresponding author on reasonable request.

## Supplementary Materials

The following supporting information can be downloaded at: https://www.mdpi.com/article/10.3390/jcm12031125/s1, Video S1: Video showing successful endoscopic treatment of a patient with a laterally extended walled-off necrosis (WON). Video showing the successful endoscopic treatment of a patient with a laterally extended walled-off necrosis (WON). Endoscopic ultrasound-guided PFC drainage using a novel lumen-opposing metal stent (LAMS) (Niti-S HOT SPAXUSTM, Taewoong Medical, Gyeonggi-do, Korea) was performed in a patient with laterally extended WON. After full expansion of the LAMS, direct endoscopic necrosectomy (DEN) was repeated using snare and forceps. At the end of each DEN session, three long plastic stents with a double-pigtail configuration were inserted into the deep portion of the WON until the next session of DEN. Following 16 sessions of DEN, the plastic stents and LAMS were removed endoscopically after confirming complete improvement.
